# Supplementary material for: Development of chemokine network inhibitors using combinatorial saturation mutagenesis
Source: Commun Biol. 2025 Apr 3;8:549. doi: 10.1038/s42003-025-07778-6 (PMC11969024; doi:10.1038/s42003-025-07778-6)
Supplement: Supplementary file 4 — Supplementary Data 2 [file 42003_2025_7778_MOESM4_ESM.zip › SupplementaryData3/scripts/xattune_dr.tar!/./script/analysis/JK/JK_E182_CXCL11_ATC_plot.pdf]

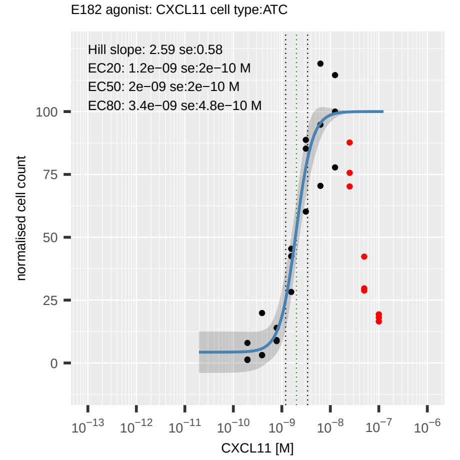

E182 agonist: CXCL11 cell type:ATC
Hill slope: 2.59 se:0.58
EC20: 1.2e−09 se:2e−10 M
EC50: 2e−09 se:2e−10 M
EC80: 3.4e−09 se:4.8e−10 M
normalised cell count
0
25
50
75
100
$10^{-13}$
$10^{-12}$
$10^{-11}$
$10^{-10}$
$10^{-9}$
$10^{-8}$
$10^{-7}$
$10^{-6}$
CXCL11 [M]
